# Brevicidine acts as an effective sensitizer of outer membrane-impermeable conventional antibiotics for *Acinetobacter baumannii* treatment

**DOI:** 10.3389/fmicb.2023.1304198

**Published:** 2023-12-15

**Authors:** Xinyi Zhong, Kai Deng, Xiuhan Yang, Xu Song, Yuanfeng Zou, Xun Zhou, Huaqiao Tang, Lixia Li, Yuping Fu, Zhongqiong Yin, Hongping Wan, Xinghong Zhao

**Affiliations:** ^1^Center for Sustainable Antimicrobials, Department of Pharmacy, Sichuan Agricultural University, Chengdu, China; ^2^Center for Infectious Diseases Control (CIDC), Sichuan Agricultural University, Chengdu, China; ^3^Key Laboratory of Animal Disease and Human Health of Sichuan Province, Sichuan Agricultural University, Chengdu, China

**Keywords:** antibiotic-resistant, brevicidine, synergistic effects, *Acinetobacter baumannii*, bacteraemia model

## Abstract

The antibiotic resistance of *Acinetobacter baumannii* poses a significant threat to global public health, especially those strains that are resistant to carbapenems. Therefore, novel strategies are desperately needed for the treatment of infections caused by antibiotic-resistant *A. baumannii*. In this study, we report that brevicidine, a bacterial non-ribosomally produced cyclic lipopeptide, shows synergistic effects with multiple outer membrane-impermeable conventional antibiotics against *A. baumannii*. In particular, brevicidine, at a concentration of 1 μM, lowered the minimum inhibitory concentration of erythromycin, azithromycin, and rifampicin against *A. baumannii* strains by 32–128-fold. Furthermore, mechanistic studies were performed by employing erythromycin as an example of an outer membrane-impermeable conventional antibiotic, which showed the best synergistic effects with brevicidine against the tested *A. baumannii* strains in the present study. The results demonstrate that brevicidine disrupted the outer membrane of *A. baumannii* at a concentration range of 0.125–4 μM in a dose-dependent manner. This capacity of brevicidine could help the tested outer membrane-impermeable antibiotics enter *A. baumannii* cells and thereafter exert their antimicrobial activity. In addition, the results show that brevicidine–erythromycin combination exerted strong *A. baumannii* killing capacity by the enhanced inhibition of adenosine triphosphate biosynthesis and accumulation of reactive oxygen species, which are the main mechanisms causing the death of bacteria. Interestingly, brevicidine and erythromycin combination showed good therapeutic effects on *A. baumannii*-induced mouse peritonitis–sepsis models. These findings demonstrate that brevicidine is a promising sensitizer candidate of outer membrane-impermeable conventional antibiotics for treating *A. baumannii* infections in the post-antibiotic age.

## Introduction

*Acinetobacter baumannii* is a nosocomial Gram-negative pathogen that is responsible for hospital-acquired infections, such as respiratory tract infections, bacteremia, urinary tract infections, surgical wound infections, and meningitis (Peleg et al., 2008; Fishbain and Peleg, 2010; Whiteway et al., 2022). Resistance to the last-resort antibiotic carbapenem makes this bacterium an urgent threat to public health and a member of the most problematic nosocomial ESKAPE pathogens. Carbapenem-resistant *A. baumannii* has been classified by the World Health Organization (WHO) as a class of bacterium for which research and development of new therapeutic strategies are critically needed (Tacconelli et al., 2018). Unfortunately, the fact is that the number of newly approved first-in-class antibiotics has been steadily decreasing in the past two decades, especially the number of antibiotics for the treatment of infections caused by Gram-negative pathogens (Batta et al., 2020; Brown and Wobst, 2021; Butler et al., 2023). Therefore, novel therapeutic strategies are desperately needed for the treatment of infections caused by antibiotic-resistant *A. baumannii*. The development of antibiotic sensitizers is an effective strategy to restore the antimicrobial activity of antibiotics against resistant pathogens.

Brevicidine (Bre), a bacterial non-ribosomally produced cyclic lipopeptide, was found in *Brevibacillus laterosporus* DSM25 by genome mining. Previous studies have shown that brevicidine has potent and selective antimicrobial activity against gram-negative pathogens, including *Enterobacter cloacae, Escherichia coli, Pseudomonas aeruginosa*, and *Klebsiella pneumoniae* (Li et al., 2018; Zhao et al., 2020a; Zhao and Kuipers, 2021a), which are members of the critical pathogens listed by WHO (Tacconelli et al., 2018). However, brevicidine showed much less antimicrobial activity against *Acinetobacter baumannii*, which is an important member of the critical pathogens listed by WHO (Li et al., 2018; Tacconelli et al., 2018; Zhao and Kuipers, 2021a). Our previous study demonstrates that brevicidine exerts its potent bactericidal activity against *E. coli* by interacting with LPS on the outer membrane and targeting phosphatidylglycerol and cardiolipin on the inner membrane, thereby dissipating the proton motive force of bacteria, which results in metabolic perturbations (Zhao et al., 2023). Considering a different mechanism of action of brevicidine from conventional antibiotics, we hypothesized that it could serve as an antibiotic sensitizer for conventional antibiotics in controlling infections caused by *A. baumannii*.

In this study, we evaluated the antimicrobial activity of 11 conventional antibiotics in combination with brevicidine against antibiotic-resistant *A. baumannii*. The results show that brevicidine exhibited synergistic effects with multiple outer membrane-impermeable conventional antibiotics, including erythromycin, azithromycin, rifampicin, vancomycin, and meropenem, against the tested antibiotic-resistant *A. baumannii* strains. Subsequently, the mechanism by which brevicidine sensitizes *A. baumannii* to outer membrane-impermeable conventional antibiotics was investigated by employing multiple fluorescent probes. The results demonstrate that brevicidine sensitizes antibiotic-resistant *A. baumannii* to outer membrane-impermeable conventional antibiotics by disrupting the bacterial outer membrane and thereafter promotes the entry of antibiotics for treating the pathogens, which results in enhanced metabolic perturbations, including the accumulation of reactive oxygen species (ROS) in bacteria and inhibition of adenosine triphosphate (ATP) synthesis. Finally, the mouse peritonitis–sepsis models demonstrate that brevicidine has potent synergistic effects with erythromycin, a member of outer membrane-impermeable conventional antibiotics, *in vivo*. This study provides an alternative antibiotic sensitizer for controlling infections caused by the critical antibiotic-resistant pathogen, carbapenem-resistant *A. baumannii*.

## Materials and methods

### Ethical statement

All animal experiments conformed to the Guide for the Care and Use of Laboratory Animals from the National Institutes of Health, and all procedures were approved by the Animal Research Committee of Sichuan Agricultural University, Sichuan, China.

### Purification of brevicidine

Methods for the purification of brevicidine have been described in detail in a previous study (Zhao et al., 2020a), and the purity of purified brevicidine in trace amounts was analyzed by high-performance liquid chromatography, which showed more than 99% purity (Zhao et al., 2023). Brevicidine was dissolved in Mili-Q water at a concentration of 2.56 mM as the mother solution.

### Antibiotics used in this study

Nisin (CAS#: 1414-45-5, ≥95%) was purchased from Handary S.A. (Brussels, Belgium); tetracycline (CAS#: 64-75-5, ≥900 mcg/mg), amikacin (CAS#: 39831-55-5, ≥98%), erythromycin (CAS#: 114-07-8, 850 μg/mg), azithromycin (CAS#: 83905-01-5, ≥98%), rifampicin (CAS#: 13292-46-1, ≥97%), penicillin G (CAS#: 113-98-4, ≥1500 μ/mg), and ampicillin (CAS#: 69-52-3, ≥98%) were purchased from Hefei Bomei Biotechnology Co., Ltd (China); vancomycin (CAS#: 1404-93-9, 900 μg/mg) was purchased from Shanghai Aladdin Biochemical Technology Co., Ltd (China); and meropenem (CAS#: 119478-59-7, ≥98%) was purchased from Shanghai Macklin Biochemical Co., Ltd (China). Polymyxin B (CAS#: 1405-20-5, ≥6,000 μ/mg) was purchased from Beijing Solarbio Science & Technology Co., Ltd (China). Tetracycline, erythromycin, azithromycin, rifampicin, vancomycin, penicillin G, and meropenem were dissolved in dimethyl sulfoxide (DMSO) at a concentration of 25.6 mM for preparing the mother solution, while amikacin, nisin, polymyxin B, and ampicillin were dissolved in Mili-Q water at a concentration of 25.6 mM for preparing the mother solution. The final concentration of DMSO in the testing culture is 1% or less (v/v), which is safe for the growth of bacteria. In addition, the control experiments were treated with the relevant solvent as controls.

### Bacterial strains used and growth conditions

*A. baumannii* ATCC 17978 and *A. baumannii* ATCC19606 were purchased from American Type Culture Collection (ATCC). The clinical *A. baumannii* strain was isolated from Chengdu, Sichuan Province of China. All bacterial strains were inoculated in LB and incubated at 37°C with aeration at 220 revolutions per minute (rpm) for preparing the overnight cultures.

### Synergy assay

The synergistic effect of brevicidine with conventional antibiotics was monitored by employing a MIC assay as the method described in previous studies (Wiegand et al., 2008; Zhao and Kuipers, 2021b; Ding et al., 2023). In brief, antibiotics were 2-fold serially diluted, while brevicidine was added at a certain concentration. Indicator strains were added at a final concentration of 5 × 10^5^ cfu/ml. After incubation at 37°C for 20 h, the OD_600_ of plates was determined. The fractional inhibitory concentration index (FICI) was calculated using the following formula: FICI = (MIC brevicidine in combination with antibiotic/MIC brevicidine) + (MIC antibiotic in combination with brevicidine/MIC antibiotic). The FICI value suggests synergistic ( ≤ 0.5), addictive (> 0.5–1), no interaction (1–4), and antagonism (> 4) effects of the two compounds (Doern, 2014).

### Outer membrane permeability assay

This assay was performed according to the procedure described in previous studies (Song et al., 2020; Xia et al., 2021). To investigate the influence of brevicidine on the integrity of the outer membrane, the fluorescent probe N-phenyl-1-naphthylamine (NPN, Aladdin) was employed. A fresh culture of *A. baumannii* ATCC 17978 was pelleted at 4,000 g for 5 min and washed three times with 10 mM HEPES containing 10 mM glucose (GHEPES, pH 7.2). After normalization of the cell density to an OD_600_ of 0.2 in GHEPES, NPN was added at a final concentration of 30 μM and incubated for 30 min in the dark for probe fluorescence to stabilize. After the cell suspension (190 μ) was added to a 96-well microplate, compounds (10 μl) were added, with the antimicrobials added after approximately 20 s, and fluorescence was monitored for 25 min. Fluorescence was recorded by using a Thermo Scientific Varioskan Flash spectral scanning multimode microplate reader with an excitation wavelength of 350 nm and an emission wavelength of 420 nm.

### Membrane integrity assay

This assay was performed according to the procedure described in a previous study (Zhao et al., 2020b). A fresh culture of *A. baumannii* ATCC 17978 was pelleted at 4,000 g for 5 min and washed three times with Mueller Hinton Broth (MHB). After normalization of the cell density to an OD_600_ of 0.2, propidium iodide was loaded at a final concentration 2.5 μg/ml and incubated for 10 min in the dark for probe fluorescence to stabilize. After the cell suspension was added to a 96-well microplate, brevicidine (0.125–4 μM), tetracycline (2 μM), or polymyxin B (2 μM) were added, with the antimicrobials added after approximately 20 s, and fluorescence was monitored for 120 min. Fluorescence was recorded by using a Thermo Scientific Varioskan Flash multimode microplate reader with an excitation wavelength of 533 nm and an emission wavelength of 617 nm.

### DiSC_3_(5) assay

*A. baumannii* ATCC 17978 was grown to an OD_600_ of 0.8. The culture was pelleted at 4,000 × g for 5 min and washed three times with MHB. The cell density was normalized to an OD_600_ of 0.2, loaded with 2 μM DiSC_3_(5) dye, and incubated for 30 min in the dark for probe fluorescence to stabilize. After incubation, the cell suspension was added to a 96-well microplate and incubated for 15 min. After that, the cells were treated with brevicidine (0.125–4 μM), tetracycline (2 μM), or polymyxin B (2 μM). Fluorescence was monitored for 55 min, with the compounds added after approximately 20 s. Fluorescence was recorded by using a Thermo Scientific Varioskan Flash multimode microplate reader with an excitation wavelength of 622 nm and an emission wavelength of 670 nm.

### Fluorescence microscopy assay

*A. baumannii* ATCC 17978 was grown to an OD_600_ of 0.8 in MHB. The culture was pelleted at 4,000 g for 5 min and washed three times with MHB. After normalization of the cell density to an OD_600_ of 0.2 in MHB, *A. baumannii* cells were then challenged with brevicidine (4 μM), brevicidine (2 μM), brevicidine (1 μM), erythromycin (0.25 μM), brevicidine (4 μM) plus erythromycin (0.25 μM), brevicidine (2 μM) plus erythromycin (0.125 μM), brevicidine (1 μM) plus erythromycin (0.625 μM), tetracycline (2 μM), or polymyxin B (2 μM). After incubation at 37°C for 5 min, cells were collected by centrifugation. Subsequently, NucGreen and EthD-III (LIVE/DEAD Bacterial Viability Kit, Solarbio, catalog no. EX3000) were added to the above cells. After incubation at room temperature for 15 min, cells were washed three times with MHB. Then, the cell suspensions were loaded on 1.5% agarose pads and analyzed by a Nikon 80i microscope (Japan).

### Time-killing assay

This assay was performed according to a previously described procedure (Ling et al., 2015; Zhao and Kuipers, 2021c; Zhao et al., 2021b,c; Zhan et al., 2023). An overnight culture of *A. baumannii* ATCC 17978 was diluted 50-fold in MHB and incubated at 37°C with aeration at 220 rpm. Bacteria were grown to an OD_600_ of 0.8, and then, the concentration of cells was adjusted to ≈1 × 10^7^ cells per ml. *A. baumannii* were then challenged with brevicidine (4 μM), brevicidine (2 μM), brevicidine (1 μM), erythromycin (0.25 μM), brevicidine (4 μM) plus erythromycin (0.25 μM), brevicidine (2 μM) plus erythromycin (0.125 μM), brevicidine (1 μM) plus erythromycin (0.625 μM), or polymyxin B (4 μM) in culture tubes at 37°C and 220 rpm. Non-treated *A. baumannii* was used as untreated control. At desired time points, 200 μl aliquots were taken, centrifuged at 6,000 g for 5 min, and resuspended in 200 μl of MHB. Overall, 10-fold serially diluted samples were plated on MHA plates. After incubation at 37°C overnight, colonies were counted and c.f.u. per mL was calculated.

### ATP measurement

The intracellular ATP levels were measured using a commercial Enhanced ATP Assay Kit (Beyotime, catalog no. S0027). A fresh culture of *A. baumannii* was pelleted at 4,000 g for 5 min and washed three times with MHB. The cell density was normalized to an OD_600_ of 0.2 and loaded with different components. At 1 h post treatment, cells were collected and lysed with grinding beads. After centrifugation at 12,000 g for 10 min, 100 μl of the supernatant was taken out, mixed with 100 μl of detecting solution, and incubated for 5 min at room temperature. Luminescence was measured with a Thermo Scientific Varioskan Flash multimode microplate reader. Carbonyl cyanide m-chlorophenyl hydrazone (CCCP, Sigma–Aldrich, CAS:555-60-2) (20 μg/ml) was used as a positive control. The relative ATP levels were calculated using the measured luminescence values vs. the luminescence value of untreated cells.

### Determination of reactive oxygen species (ROS)

The levels of ROS in *A. baumannii* treated with different components were measured by employing the fluorescent probe 2′,7′-dichlorofluorescein diacetate (DCFH-DA) (Hu et al., 2023; Li et al., 2023), following the manufacturer's instruction (Beyotime, catalog no. S0033S). In brief, a fresh culture of *A. baumannii* was pelleted at 4,000 g for 5 min and washed three times with MHB. The cell density was normalized to an OD_600_ of 1.0 and loaded with DCFH-DA at a final concentration of 10 μM, and the mixture was incubated at 37°C for 30 min. After washing three times with MHB, 190 μl of probe-labeled bacterial cells, followed by 10 μl of different components, were added to a 96-well plate. Fluorescence was recorded by using a Thermo Scientific Varioskan Flash multimode microplate reader with the excitation wavelength of 488 nm and the emission wavelength of 525 nm. The antioxidant N-acetyl-L-cysteine (NAC, 6 mM) was used as a control to neutralize the production of ROS.

### Mouse peritonitis–sepsis models

To assess the *in vivo* bioavailability of brevicidine and erythromycin combination in *A. baumannii*-induced mouse peritonitis–sepsis model, 70 BALB/c male mice (n=10 per group) were infected intraperitoneally with *A. baumannii* (ATCC 17978) at a dose of 2 × 10^9^ c.f.u. per mouse that leads to 80% of death. At 1 h post-infection, mice were treated with brevicidine (5 mg/kg), erythromycin (5 mg/kg), brevicidine (5 mg/kg) plus erythromycin (1.25 mg/kg), brevicidine (5 mg/kg) plus erythromycin (2.5 mg/kg), brevicidine (5 mg/kg) plus erythromycin (5 mg/kg), or 0.9% NaCl *via* intravenous injection. Mice without *A. baumannii* infections were used as the non-infection control. The survival rates of different groups were monitored for 7 days.

To gain a deeper insight into the synergistic effect of brevicidine and erythromycin *in vivo*, 36 BALB/c male mice (*n* = 6 per group) were infected intraperitoneally with *A. baumannii* (ATCC 17978) at a dose of 1 × 10^9^ c.f.u. per mouse that does not lead to death. At 1 h post-infection, mice were treated with brevicidine (5 mg/kg), erythromycin (5 mg/kg), brevicidine (5 mg/kg) plus erythromycin (1.25 mg/kg), brevicidine (5 mg/kg) plus erythromycin (2.5 mg/kg), brevicidine (5 mg/kg) plus erythromycin (5 mg/kg), or 0.9% NaCl *via* intravenous injection. Mice without *A. baumannii* infections were used as the non-infection control. At 24 h post-infection, organs, including the heart, liver, spleen, lung, and kidney, were collected to measure the bacterial load.

### Statistical analysis

**Figure 1:**
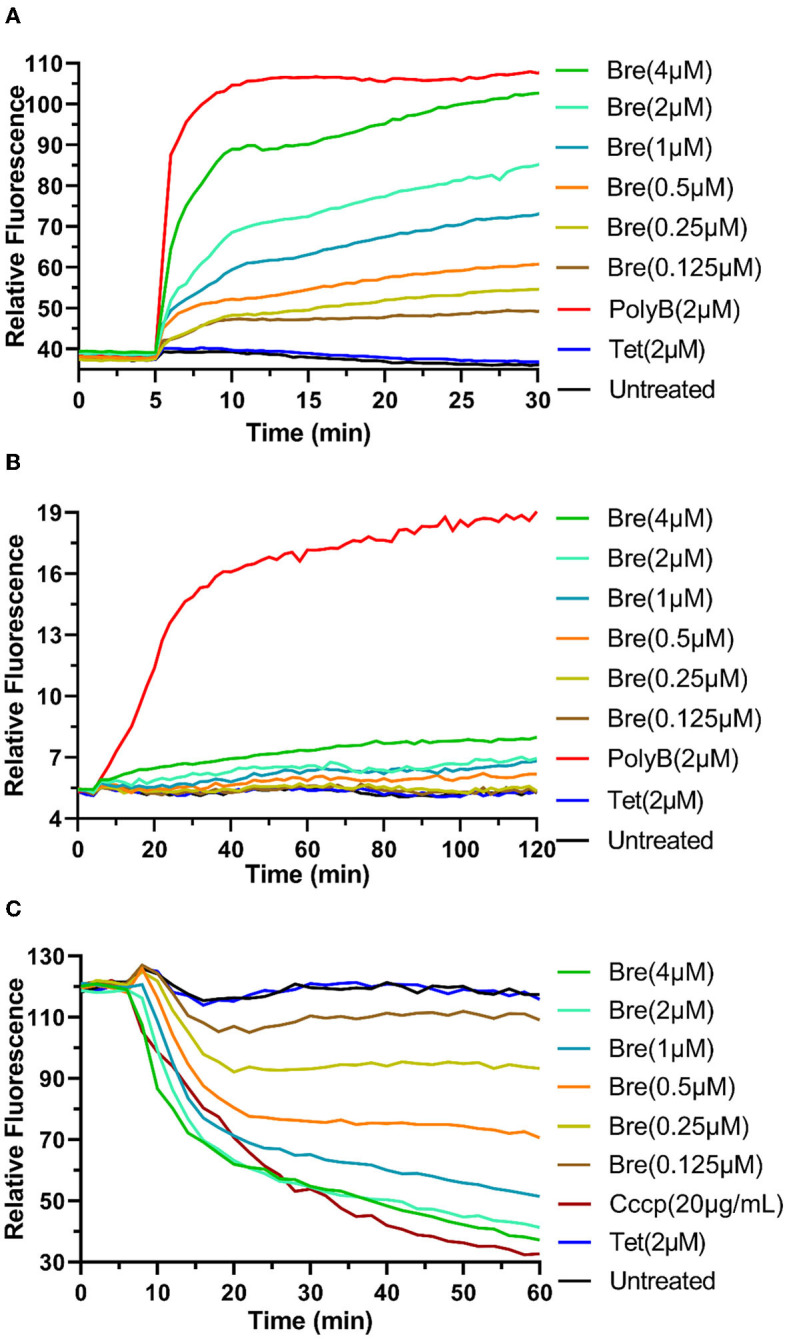
Brevicidine sensitizes *A. baumannii* to conventional antibiotics by disrupting the outer membrane and proton motive force. **(A)** NPN fluorescence in *A. baumannii* ATCC17978 cells upon exposure to brevicidine at a concentration range of 0.125–4 μM. PolyB and Tet were used as outer membrane disruption and non-outer membrane disruption antibiotic controls, respectively. Data are presented as means (*n* = 3). **(B)** *A. baumannii* ATCC17978 cells pretreated with propidium iodide were exposed to brevicidine at a concentration range of 0.125–4 μM, and the extent of membrane leakage was visualized as an increase in fluorescence. PolyB and Tet were used as membrane disruption and non-membrane disruption antibiotic controls, respectively. Data are presented as means (*n* = 3). **(C)** DiSC_3_(5) fluorescence in *A. baumannii* ATCC17978 cells upon exposure to brevicidine at a concentration range of 0.125–4 μM. Carbonyl cyanide m-chlorophenyl hydrazone (CCCP) and Tet were used as proton motive force disruption and non-proton motive force disruption compound controls, respectively. Data are presented as means (*n* = 3).

GraphPad Prism 8.0 was used to fit the data in Figures 1, **3**, **4B**–**G**. The statistical significance of the data was assessed using a two-tailed Student's *t*-test with GraphPad Prism 8.0. Correlation analyses were evaluated by Pearson *r*^2^ test, ns: *p* > 0.05, ^*^p < 0.05, ^**^p < 0.01, ^***^p < 0.001, and ^****^p < 0.0001.

## Results and discussion

### Brevicidine shows good synergistic effects with outer membrane-impermeable conventional antibiotics against *A. baumannii*

To assess the synergistic effects of brevicidine with 11 conventional antibiotics, a synergy assay was performed according to the method described in previous studies with mild modifications (Wiegand et al., 2008; Cochrane and Vederas, 2014; Zhao et al., 2021a). Nisin and vancomycin are lipid II-targeting antibiotics that show potent antimicrobial activity against Gram-positive pathogens (Breukink et al., 2003), while they are less active against most Gram-negative pathogens due to the outer membrane barrier. Rifampicin is an RNA-inhibiting antibiotic and is usually used in combination with other antibiotics (Campbell et al., 2001). Erythromycin (Ery) and azithromycin are antibiotics that exert their bacteriostatic antimicrobial activity by targeting the bacterial 50S ribosomal subunit (Tanaka et al., 1973; Parnham et al., 2014), while tetracycline (Tet) and amikacin are antibiotics that exert their antimicrobial activity by targeting the bacterial 30S ribosomal subunit (Speer et al., 1992; Alangaden et al., 1998). Penicillin G, ampicillin, and meropenem are bacteria cell wall synthesis inhibitors, and these antibiotics have good antimicrobial activity against gram-negative pathogens; however, bacterial resistance is common for these antibiotics (Sumita et al., 1992; Sugimoto et al., 2002; Ono et al., 2005). Polymyxin B (PolyB) is a bactericidal antibiotic that targets the Gram-negative bacteria envelope (Trimble et al., 2016).

The synergy test results showed that brevicidine has synergistic effects with tested outer membrane-impermeable conventional antibiotics against *A. baumannii* (Tables 1–**3**). The increasing synergistic effects were observed with erythromycin, showing a decrease in minimum inhibitory concentration (MIC) of 128-fold against all tested *A. baumannii* strains, including a clinically isolated carbapenem-resistant *A. baumannii* strain (Tables 1–**3**). A previous study showed that a cathelicidin-derived membrane-targeting peptide D-11 has shown synergistic effects with erythromycin against *Klebsiella pneumoniae*; the MIC of erythromycin decreased 64-fold, from 128 μM to 4 μM, in the presence of D-11 at a concentration of 4 μM (Cebrián et al., 2021). In this regard, brevicidine is much more capable than D-11 because the MIC of erythromycin decreased 128-fold, from 8/16 μM to 0.0625/0.125 μM, in the presence of brevicidine at a concentration of 1/2 μM. Strong synergistic effects were also found between brevicidine and rifampicin/azithromycin, with a decrease in MIC of 32–128-fold against tested *A. baumannii* strains (Tables 1–**3**). This response is expected because rifampicin and azithromycin have shown synergistic effects with multiple membrane-active peptides (Cochrane and Vederas, 2014; Song et al., 2020; Cebrián et al., 2021; Xia et al., 2021). However, only weak synergistic effects were observed between brevicidine and the two tested anti-30S ribosomal subunit antibiotics, namely, tetracycline and amikacin.

**Table 1:** Synergy between brevicidine and antibiotics against *A. baumannii* ATCC17978.

**Family/antibiotic**	**MIC**^**a**^ **at brevicidine concentrations**^**a**^ **of**				**MMD**	**FICI**
	**0**	**0.25**	**0.5**	**1**		
**Tetracyclines**						
Tetracycline	2	2	1	1	2x	0.63
**Aminoglycosides**						
Amikacin	8	4	4	2	4x	0.5
**Macrolides**						
Erythromycin	8	1	0.5	0.06	128x	0.19
Azithromycin	16	2	1	0.5	32x	0.19
**Rifamycins**						
Rifampicin	2	1	0.25	0.06	32x	0.25
**Lipid II targeting peptide antibiotics**						
Vancomycin	32	16	8	4	8x	0.38
Nisin	4	2	2	0.5	8x	0.38
β**-Lactams**						
Penicillin G	128	128	64	64	2x	0.63
Ampicillin	256	256	128	128	2x	0.63
Meropenem	4	2	0.5	0.5	8x	0.25
**Polymyxins**						
Polymyxin B	1	1	1	1	0	1.25

MIC, minimum inhibitory concentration; MMD, maximum MIC decrease; FICI, fractional inhibitory concentration index [FICI = (MIC brevicidine in combination with antibiotic/MIC brevicidine) + (MIC antibiotic in combination with brevicidine/MIC antibiotic)].

^a^all values reported in μM. Brevicidine showed a MIC of 4 μM against A. baumannii ATCC17978.

Moderate synergistic effects were observed between brevicidine and vancomycin against two *A. baumannii* reference strains (ATCC17978 and ATCC19606), with a decrease in MIC of 8-fold (Tables 1, 2). Notably, the antimicrobial activity of vancomycin against the clinically isolated carbapenem-resistant *A. baumannii* strain increased 64-fold in the presence of brevicidine at a concentration of 2 μM (Table 3). Interestingly, the antimicrobial activity of nisin increased 8-fold in the presence of brevicidine, which was unexpected due to its large molecular size (3.4 kDa). Li et al. reported that a series of outer-membrane-acting peptides have shown good synergistic effects with lipid II-targeting peptide antibiotics, vancomycin and nisin, against *A. baumannii* strains (Li et al., 2021). Our results show that brevicidine exhibited comparable synergistic effects with vancomycin than these peptides with vancomycin.

**Table 2:** Synergy between brevicidine and antibiotics against *A. baumannii* ATCC19606.

**Bacteria/antibiotic**	**MIC**^**a**^ **at brevicidine concentrations**^**a**^ **of**				**MMD**	**FICI**
	**0**	**0.25**	**0.5**	**1**		
**Tetracyclines**						
Tetracycline	2	2	2	0.5	4x	0.5
**Aminoglycosides**						
Amikacin	8	8	8	2	4x	0.5
**Macrolides**						
Erythromycin	16	2	2	0.13	128x	0.19
Azithromycin	128	16	16	1	128x	0.19
**Rifamycins**						
Rifampicin	1	0.25	0.06	0.02	64x	0.19
**Lipid II targeting peptide antibiotics**						
Vancomycin	64	16	16	4	16x	0.31
Nisin	4	2	2	0.5	8x	0.38
β**-Lactams**						
Penicillin G	256	256	256	128	2x	0.75
Ampicillin	256	256	256	256	0	1.25
Meropenem	8	2	1	1	8x	0.25
**Polymyxins**						
Polymyxin B	1	1	1	1	0	1.25

MIC, minimum inhibitory concentration; MMD, maximum MIC decrease; FICI, fractional inhibitory concentration index [FICI = (MIC brevicidine in combination with antibiotic/MIC brevicidine) + (MIC antibiotic in combination with brevicidine/MIC antibiotic)].

^a^all values reported in μM. Brevicidine showed a MIC value of 4 μM against A. baumannii ATCC19606 strains.

**Table 3:** Synergy between brevicidine and antibiotics against a clinically isolated carbapenem-resistant *A. baumannii* stain.

**Antibiotic**	**MIC**^**a**^ **at brevicidine concentrations**^**a**^ **of**				**MMD**	**FICI**
	**0**	**0.5**	**1**	**2**		
**Tetracyclines**						
Tetracycline	4	4	2	1	4x	0.5
**Aminoglycosides**						
Amikacin	8	8	4	2	4x	0.5
**Macrolides**						
Erythromycin	16	0.5	0.25	0.13	128x	0.14
Azithromycin	128	8	4	2	64x	0.16
**Rifamycins**						
Rifampicin	0.5	0.02	0.01	0.01	64x	0.14
**Lipid II targeting peptide antibiotics**						
Vancomycin	32	16	4	0.5	64x	0.25
Nisin	4	1	0.5	0.5	8x	0.25
β**-Lactams**						
Penicillin G	256	256	256	128	2x	0.75
Ampicillin	256	256	256	256	0	1.06
Meropenem	16	2	2	0.5	32x	0.19
**Polymyxins**						
Polymyxin B	1	1	1	1	0	1.25

MIC, minimum inhibitory concentration; MMD, maximum MIC decrease; FICI, fractional inhibitory concentration index [FICI = (MIC brevicidine in combination with antibiotic/MIC brevicidine) + (MIC antibiotic in combination with brevicidine/MIC antibiotic)].

^a^all values reported in μM. The MIC value of brevicidine against the tested clinically isolated carbapenem-resistant A. baumannii stain was 8 μM.

Brevicidine showed good synergistic effects with meropenem, a member of carbapenem antibiotics, against two *A. baumannii* reference strains (ATCC17978, ATCC19606), with a decrease in MIC of 8-fold (Tables 1, 2). Notably, brevicidine restored the antimicrobial activity of meropenem against a clinically isolated carbapenem-resistant *A. baumannii* strain (Table 3). However, no synergistic effects were observed with penicillin G or ampicillin because these two antibiotics are outer membrane permeable, and the strains of the above two antibiotics carry beta-lactamase. Brevicidine showed no synergistic effects with polymyxin B, which is a bactericidal antibiotic that exerts its antimicrobial activity against Gram-negative bacteria by targeting the cell envelope (Trimble et al., 2016). This can be further explained by the fluorescent probe assays. Brevicidine shows synergistic effects with some tested antibiotics due to its outer membrane disruption ability; however, polymyxin B can pass outer membrane by itself. Together, these results demonstrate that brevicidine is an effective sensitizer of *A. baumannii* to outer membrane-impermeable conventional antibiotics, such as erythromycin, azithromycin, rifampicin, vancomycin, and meropenem.

### Brevicidine sensitizes *A. baumannii* to conventional antibiotics by disrupting the outer membrane and proton motive force

To investigate the influence of brevicidine on *A. baumannii* membrane, three fluorescent probe assays were performed. N-Phenyl-1-naphthylamine (NPN) is a gram-negative bacterial impermeable fluorescent probe due to the outer membrane barrier (Helander and Mattila-Sandholm, 2000). If the integrity of the outer membrane is disrupted, this fluorescent probe can reach the phospholipid layer, resulting in a significant increase in fluorescence. The results show that the fluorescence signal was increased by the addition of brevicidine at a concentration range of 0.125–4 μM in a dose-dependent manner (Figure 1A), demonstrating that brevicidine disrupted the outer membrane of *A. baumannii* at sub-MIC concentrations. This capacity of brevicidine could promote outer membrane-impermeable antibiotics, such as erythromycin, azithromycin, and vancomycin, enter *A. baumannii* and thereafter exert their antimicrobial activity.

Propidium iodide (PI) is a membrane-permeant fluorescent probe. If the membrane integrity is disrupted, PI can enter bacteria and bind to nucleic acid, resulting in a prominent increase in fluorescence (Zhao et al., 2020b). The fluorescence signal indicated no significant change after the addition of brevicidine at a concentration range of 0.125–4 μM during 2 h of monitoring (Figure 1B), demonstrating that brevicidine sensitizes *A. baumannii* to outer membrane-impermeable conventional antibiotics *via* disrupting the cytoplasmic membrane.

Our previous study shows that brevicidine can disrupt the proton motive force of *E. coli* (Zhao and Kuipers, 2021a; Zhao et al., 2023). To test the effect of brevicidine on the proton motive force of *A. baumannii*, a DiSC_3_(5) (3,3′-dipropylthiadicarbocyanine iodide) fluorescent probe assay was performed. DiSC_3_(5) accumulates in the cytoplasmic membrane of bacteria in response to the Δψ component of the proton motive force (Wu et al., 1999; Stokes et al., 2020). When the transmembrane ΔpH potential of bacteria is disrupted, cells compensate by increasing the Δψ, resulting in enhanced DiSC_3_(5) uptake into the cytoplasmic membrane and therefore decreased fluorescence (Wu et al., 1999; Stokes et al., 2020). The fluorescence signal of DiSC_3_(5) was decreased (Figure 1C) after treatment with brevicidine at a concentration range of 0.125–4 μM in a dose-dependent manner (Figure 1C), indicating that brevicidine dissipated the proton motive force of *A. baumannii*.

**Figure 2:**
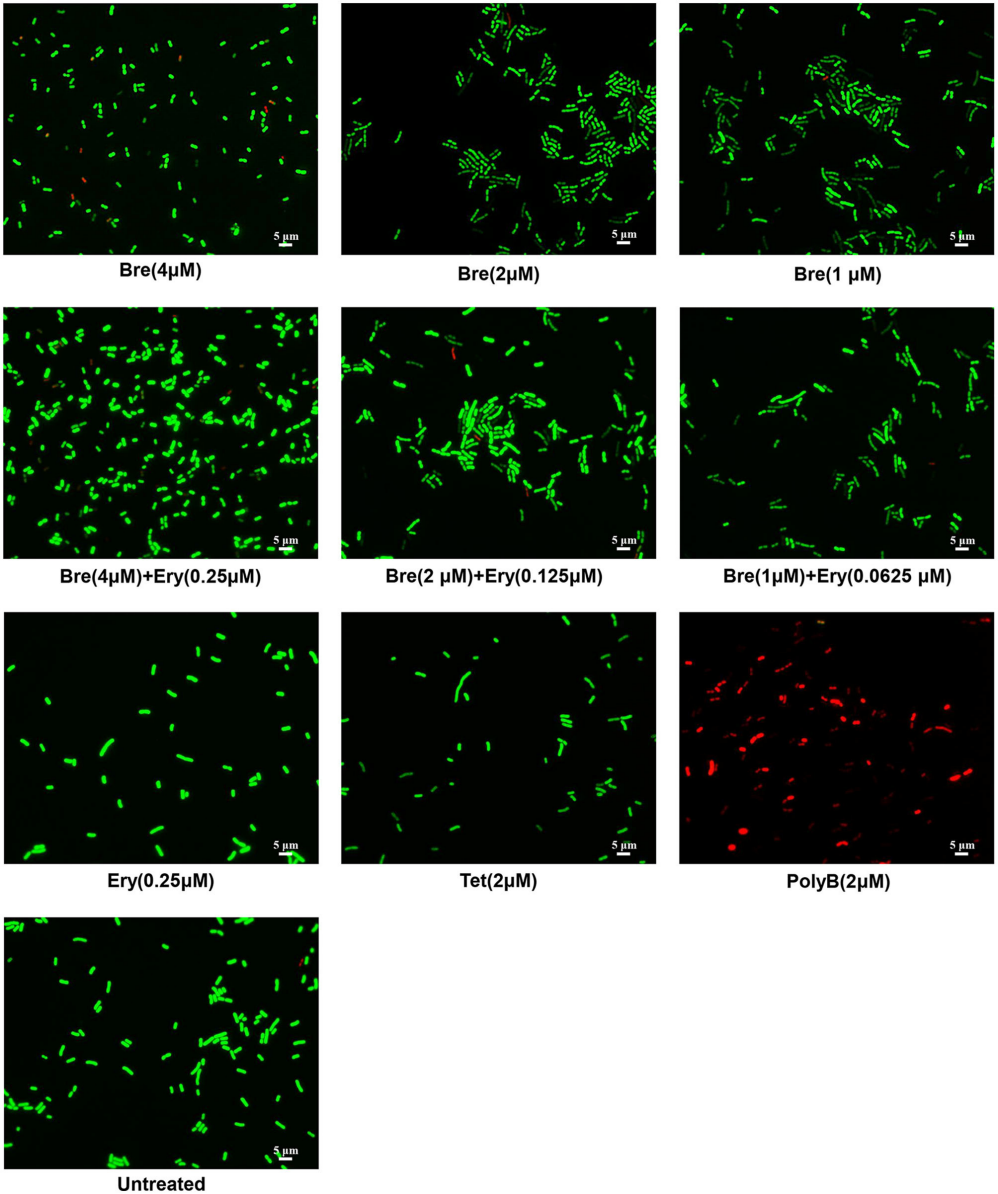
Fluorescence microscopy images of *A. baumannii* ATCC17978 cells, which were challenged with brevicidine (4 μM), brevicidine (2 μM), brevicidine (1 μM), erythromycin (0.25 μM), brevicidine (4 μM) plus erythromycin (0.25 μM), brevicidine (2 μM) plus erythromycin (0.125 μM), brevicidine (1 μM) plus erythromycin (0.625 μM), tetracycline, or polymyxin B (2 μM) for 5 min. Green denotes a cell with an intact membrane, whereas red denotes a cell with a compromised membrane.

To investigate the influence of brevicidine and outer membrane-impermeable conventional antibiotic combination on the membrane integrity of *A. baumannii*, membrane permeability assays were performed by using a commercial LIVE/DEAD Bacterial Viability kit, which contains NucGreen and EthD-III. Cells with an intact membrane will stain green, whereas cells with a compromised membrane will stain red. Polymyxin B (PolyB) was used as a membrane disruption antibiotic control, while tetracycline was used as an antibiotic control without membrane disruption. After incubation for 5 min, green cells were observed for brevicidine or brevicidine-erythromycin combination treated *A. baumannii*, demonstrating that brevicidine-erythromycin combination shows enhanced antimicrobial activity, which does not occur via disrupting the cytoplasmic membrane integrity of *A. baumannii* (Figure 2), which is consistent with the results of PI fluorescent probe assay (Figure 1B). These results demonstrate that erythromycin, a 50S ribosomal subunit targeting bacteriostatic antibiotic, did not influence the effect of brevicidine on the cytoplasmic membrane.

### Brevicidine and outer membrane-impermeable conventional antibiotic (erythromycin) combination shows augmented *A. baumannii* killing capacity *via* enhanced ATP synthesis inhibition and ROS accumulation

**Figure 3:**
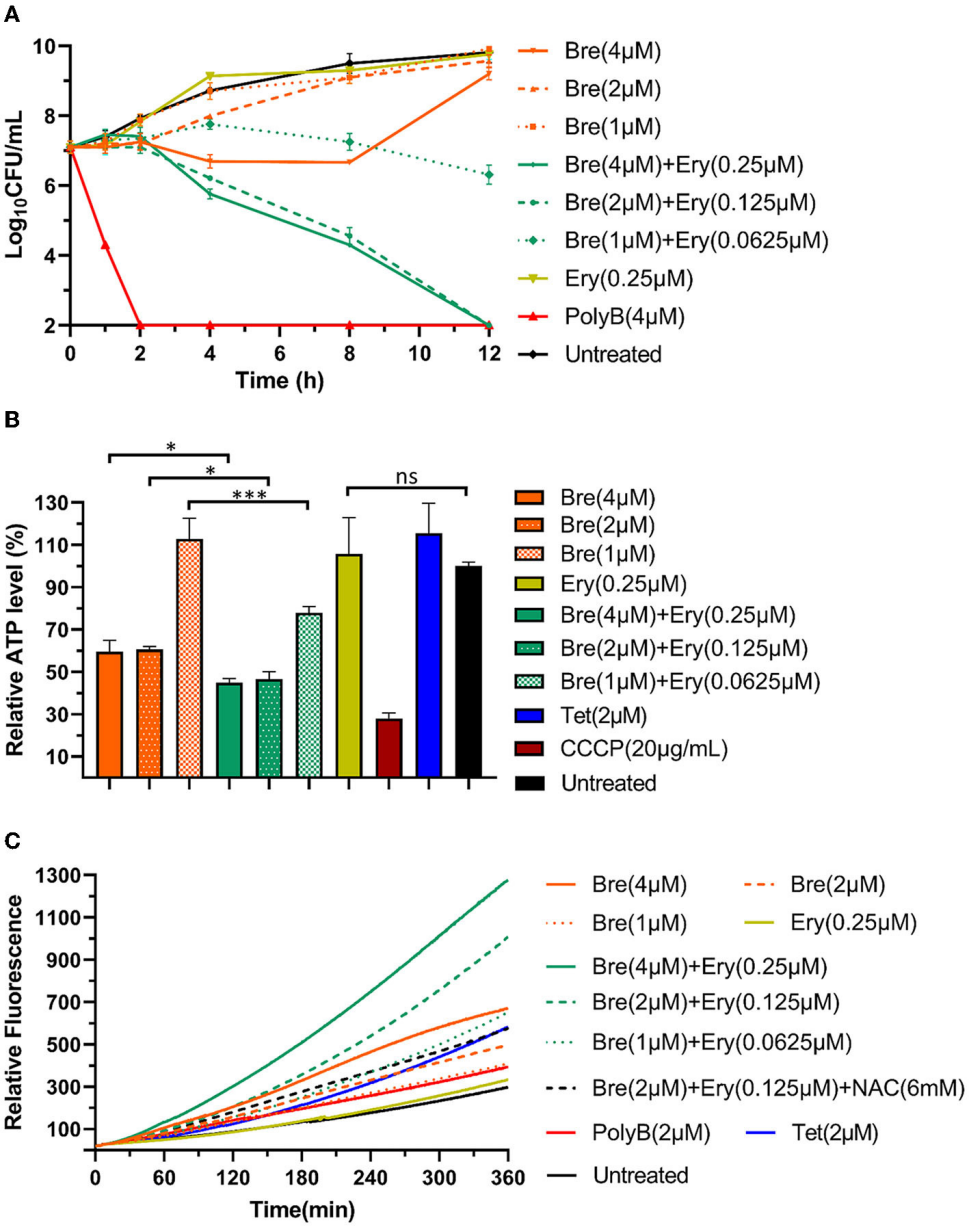
Brevicidine and outer membrane-impermeable conventional antibiotic (erythromycin) combination shows augmented *A. baumannii* killing capacity *via* enhanced inhibition of ATP synthesis and accumulation of ROS. **(A)** Time killing curve of brevicidine (4 μM), brevicidine (2 μM), brevicidine (1 μM), erythromycin (0.25 μM), brevicidine (4 μM) plus erythromycin (0.25 μM), brevicidine (2 μM) plus erythromycin (0.125 μM), brevicidine (1 μM) plus erythromycin (0.625 μM), and polymyxin B (4 μM) against *A. baumannii*. **(B)** Relative ATP concentration of *A. baumannii* cells treated with brevicidine (4 μM), brevicidine (2 μM), brevicidine (1 μM), erythromycin (0.25 μM), brevicidine (4 μM) plus erythromycin (0.25 μM), brevicidine (2 μM) plus erythromycin (0.125 μM), brevicidine (1 μM) plus erythromycin (0.625 μM), CCCP, 20 mg/ml, and tetracycline (2 μM) for 1 h. All data were presented as means ± standard deviation (*n* = 3). Correlation analyses were evaluated by Pearson *r*^2^ test. *p < 0.05; ***p < 0.001. **(C)** Accumulation of ROS in *A. baumannii* cells treated with brevicidine (4 μM), brevicidine (2 μM), brevicidine (1 μM), erythromycin (0.25 μM), brevicidine (4 μM) plus erythromycin (0.25 μM), brevicidine (2 μM) plus erythromycin (0.125 μM), brevicidine (1 μM) plus erythromycin (0.625 μM), brevicidine (2 μM) plus erythromycin (0.125 μM), N-Acetyl-L-cysteine (NAC, 6 mM), polymyxin B (2 μM), and tetracycline (2 μM). NAC, antioxidant N-Acetyl-L-cysteine.

To assess the killing capacity of brevicidine and outer membrane-impermeable conventional antibiotic (erythromycin) combination, a time-killing assay was performed as the method described in previous studies (Ling et al., 2015; Zhao et al., 2021b,c). The results show that brevicidine and erythromycin combination significantly enhanced the killing capacity of each against *A. baumannii* (Figure 3A).

The results shown in Figure 1C indicate that brevicidine dissipated the proton motive force of *A. baumannii*. The proton motive force is essential for the generation of ATP, which is an essential bioactive compound for live bacteria (Bakker and Mangerich, 1981; Ahmed and Booth, 1983; Li et al., 2020). The dissipation of the proton motive force of *A. baumannii* will inhibit or even terminate the process of ATP biosynthesis. To investigate the effect of brevicidine and erythromycin combination on the ATP level of *A. baumannii*, the intracellular ATP levels of different treatments were measured by a commercial Enhanced ATP Assay Kit (Beyotime, catalog no. S0027). Compared with untreated cells, the ATP levels of 4 and 2 μM brevicidine-treated cells were significantly decreased (Figure 3B). Erythromycin did not influence the ATP level of *A. baumannii* at a concentration of 0.25 μM, expectably, because this concentration is much lower than its MIC. Surprisingly, the ATP synthesis inhibition capacity of brevicidine was significantly enhanced in combination with erythromycin at relatively low concentrations (0.0625–0.25 μM) (Figure 3B). This effect might be contributed to the protein synthesis ability of erythromycin. Two previously reported membrane-active peptide antibiotic sensitizers, namely, SLAP-S25 and D-11, have also shown ATP synthesis inhibition activity (Song et al., 2020; Xia et al., 2021). However, these studies failed to investigate if relative antibiotics would enhance the ATP synthesis inhibition activity of these membrane-active peptide antibiotic sensitizers.

The proton motive force plays a vital role in the removal of reactive oxygen species (ROS) in bacteria (Berry et al., 2018; Zhao et al., 2023). The accumulation of ROS is an important mechanism in which antimicrobials exert their bacterial killing capacity (Guridi et al., 2015; Clauss-Lendzian et al., 2018; Yu et al., 2020; Liu et al., 2023). To determine the intracellular ROS levels of *A. baumannii* after treatment with different concentrations of brevicidine or brevicidine–erythromycin combination, a 2′,7′-dichlorofluorescein diacetate (DCFH-DA) fluorescent probe-based assay was employed (Zhao and Kuipers, 2021a; Zhao et al., 2023). DCFH-DA is a bacterial cell-permeable non-fluorescent probe. This molecule can be de-esterified intracellularly, and the de-esterified product turns to highly fluorescent 2′,7′-dichlorofluorescein upon oxidation by ROS. The results show that brevicidine caused ROS accumulation in a dose-dependent manner (Figure 3C). However, erythromycin did not cause ROS accumulation at a concentration of 0.25 μM. Interestingly, the ROS accumulation capacity of brevicidine was significantly enhanced in combination with erythromycin at relatively low concentrations (0.0625–0.25 μM) (Figure 3C), which is reasonably an important synergistic mechanism of brevicidine and erythromycin.

### Brevicidine and outer membrane-impermeable conventional antibiotic (erythromycin) combination shows good therapeutic effects in mouse peritonitis–sepsis models

**Figure 4:**
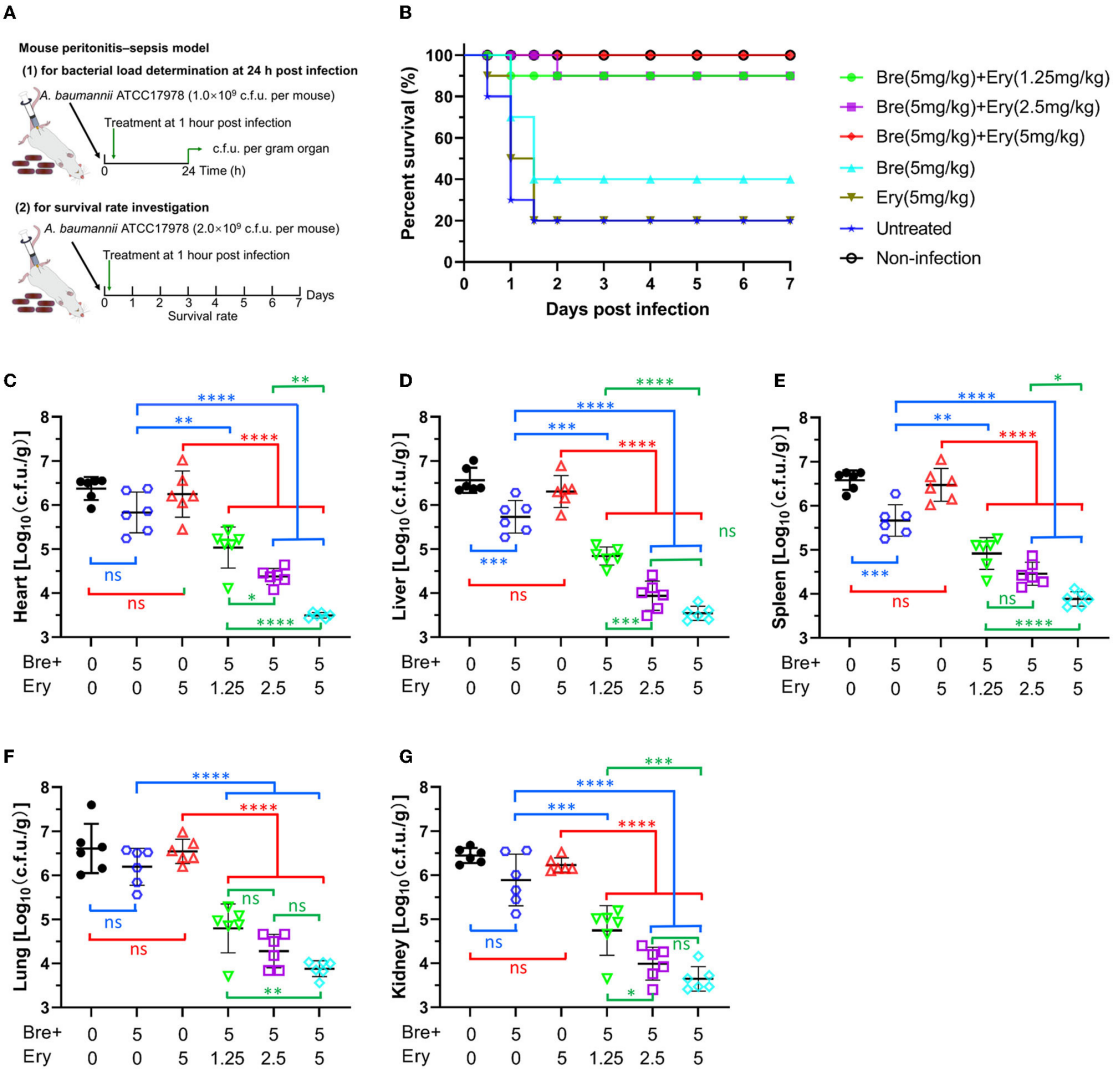
Brevicidine shows a good synergistic effect with conventional antibiotic (erythromycin) in mouse peritonitis–sepsis models. **(A)** Schemes of the experimental protocol for the mouse peritonitis–sepsis models. **(B)** Survival rates of mice in the mouse peritonitis–sepsis model (*n* = 10). Increased survival rates of mice for 7 days by a dose that leads to 80% of death of *A. baumannii* (2.0 × 10^9^ c.f.u.), treated with brevicidine (5 mg/kg), brevicidine (5 mg/kg) plus erythromycin (1.25 mg/kg), brevicidine (5 mg/kg) plus erythromycin (2.5 mg/kg), or brevicidine (5 mg/kg) plus erythromycin (5 mg/kg) are shown. **(C–G)** Brevicidine and erythromycin combination significantly reduced the bacterial load of organs of the mouse peritonitis–sepsis model. At 24 h post-infection, the mice (*n* = 6) were euthanized by cervical dislocation. Bacterial loads (Log10 c.f.u. per gram of *A. baumannii*) of the heart **(C)**, liver **(D)**, spleen **(E)**, lung **(F)**, and kidney **(G)** were counted. All data were presented as means ± standard deviation (*n* = 6). Correlation analyses were evaluated by Pearson *r*^2^ test. ns, no significance; *p < 0.05; **p < 0.01; ***p < 0.001; and ****p < 0.0001.

Given the attractive synergistic effects between brevicidine and outer membrane-impermeable conventional antibiotics, we investigated the potential of brevicidine as an antibiotic sensitizer in mouse peritonitis–sepsis models (Figure 4A). To assess the protection effect of brevicidine–erythromycin combination on *A. baumannii-*induced mouse peritonitis–sepsis models, mice were infected intraperitoneally with *A. baumannii* at a dose of 2 × 10^9^ c.f.u. per mouse that leads to 80% of death. At 1 h post-infection, drugs were introduced at single intravenous doses (Figure 4A). Erythromycin showed no protective effect on *A. baumannii-*induced mouse peritonitis–sepsis models at a dose of 5 mg/kg (Figure 4B). Brevicidine reduced the death rate of *A. baumannii*-infected mice from 80% to 60%. Excitingly, brevicidine and erythromycin showed a good synergistic effect on *A. baumannii-*induced mouse peritonitis–sepsis models, and only one mouse died in the 5 mg/kg brevicidine and 2.5/1.25 mg/kg erythromycin combination-treated group. Notably, all of the mice survived under a single dose of 5 mg/kg brevicidine plus 5 mg/kg erythromycin combination treatment (Figure 4B). These results demonstrate that brevicidine–erythromycin combination has a much stronger therapeutic efficacy than either of them alone.

To get a deeper insight into the synergistic effect of brevicidine–erythromycin combination *in vivo*, mice were infected intraperitoneally with *A. baumannii* at a dose of 1 × 10^9^ c.f.u. per mouse that does not lead to death at 24 h post-infection. At 1 h post-infection, drugs were introduced at a single intravenous dose (Figure 4A). At 24 h post-infection, all mice were sacrificed, and the hearts, livers, spleens, lungs, and kidneys were harvested for bacterial load measurement. Compared with the untreated group, erythromycin (5 mg/kg) showed no significant influence on the bacterial load of all organs investigated (Figures 4C–G), which could explain why it had no protection effect on the infected mice (Figure 4B). Brevicidine reduced the bacterial load of all organs investigated, and it significantly reduced (p < 0.001) the bacterial load of the liver and spleen (Figures 4C–G), which is consistent with the finding that brevicidine slightly reduced the death rate of *A. baumannii-*infected mice (Figure 4B). Compared with the untreated group, brevicidine–erythromycin (5 mg/kg:1.25, 2.5, or 5 mg/kg) combination significantly (*p* < 0.0001) reduced the bacterial load of all organs investigated (Figures 4C–G). In addition, compared with the brevicidine alone or the erythromycin alone treated groups, brevicidine–erythromycin (5 mg/kg:1.25, 2.5, or 5 mg/kg) combination significantly (*p* < 0.01) reduced the bacterial load of all organs investigated (Figures 4C–G). Under a dose of 5 mg/kg brevicidine treatment, the bacterial load reduction capacity of brevicidine–erythromycin combination shows in an erythromycin dose-dependent manner (Figures 4C–G). Together, these findings demonstrate the potential of brevicidine as a novel antibiotic sensitizer for the treatment of difficult-to-treat *A. baumannii* infections in the post-antibiotic age.

## Conclusion

In this study, we show that brevicidine, a bacterial non-ribosomally produced cyclic lipopeptide, has a synergistic effect with multiple outer membrane-impermeable conventional antibiotics, such as erythromycin, azithromycin, rifampicin, vancomycin, and meropenem, against the tested *A. baumannii* strains, including a clinically isolated carbapenem-resistant *A. baumannii* stain. Furthermore, mechanistic studies were performed by using erythromycin as an outer membrane-impermeable antibiotic example, which showed the best synergistic effects with brevicidine against the tested *A. baumannii* strains in the present study. The results demonstrate that brevicidine can disrupt the outer membrane of *A. baumannii*, which helps the tested outer membrane-impermeable antibiotics enter *A. baumannii* cells and thereafter exert their antimicrobial activity. In addition, the results show that brevicidine–erythromycin combination has potent ATP biosynthesis inhibition and ROS accumulation capacities that are the main mechanisms causing death of bacteria. Notably, brevicidine and erythromycin showed good synergistic effects in mouse peritonitis–sepsis models. These findings demonstrate that brevicidine is a promising sensitizer candidate of outer membrane-impermeable conventional antibiotics for the treatment of *A. baumannii* infections in the post-antibiotic age.

## Data availability statement

The original contributions presented in the study are included in the article/supplementary material, further inquiries can be directed to the corresponding authors.

## Ethics statement

The animal study was approved by the Animal Research Committee of Sichuan Agricultural University. The study was conducted in accordance with the local legislation and institutional requirements.

## Author contributions

XZhon: Conceptualization, Formal analysis, Investigation, Methodology, Visualization, Writing – original draft. KD: Conceptualization, Formal analysis, Investigation, Methodology, Visualization, Writing – original draft. XY: Conceptualization, Formal analysis, Investigation, Methodology, Visualization, Writing – original draft. XS: Investigation, Validation, Visualization, Formal analysis, Writing – review & editing. YZ: Investigation, Validation, Visualization, Methodology, Writing – review & editing. XZhou: Investigation, Validation, Visualization, Methodology, Writing – review & editing. HT: Investigation, Validation, Visualization, Formal analysis, Writing – review & editing. LL: Investigation, Validation, Visualization, Methodology, Writing – review & editing. YF: Investigation, Validation, Methodology, Writing – review & editing. ZY: Investigation, Supervision, Visualization, Methodology, Writing – review & editing. HW: Conceptualization, Supervision, Writing – original draft, Writing – review & editing. XZha: Conceptualization, Supervision, Writing – original draft, Writing – review & editing.
